# Cutting-edge technologies in neural regeneration

**DOI:** 10.1186/s13619-025-00260-y

**Published:** 2025-09-05

**Authors:** Chang-Ping Li, Ying-Ying Wang, Ching-Wei Zhou, Chen-Yun Ding, Peng Teng, Rui Nie, Shu-Guang Yang

**Affiliations:** Center for Translational Neural Regeneration Research, Sir Run Run Shaw Hospital, Zhejiang University School of Medicine, Hangzhou, Zhejiang 310016 China

**Keywords:** Neural regeneration, Optogenetics, Chemogenetics, 3D cell culture, Organoid, Gene editing, Single-cell sequencing, 3D imaging

## Abstract

Neural regeneration stands at the forefront of neuroscience, aiming to repair and restore function to damaged neural tissues, particularly within the central nervous system (CNS), where regenerative capacity is inherently limited. However, recent breakthroughs in biotechnology, especially the revolutions in genetic engineering, materials science, multi-omics, and imaging, have promoted the development of neural regeneration. This review highlights the latest cutting-edge technologies driving progress in the field, including optogenetics, chemogenetics, three-dimensional (3D) culture models, gene editing, single-cell sequencing, and 3D imaging. Prospectively, the advancements in artificial intelligence (AI), high-throughput in vivo screening, and brain-computer interface (BCI) technologies promise to accelerate discoveries in neural regeneration further, paving the way for more precise, efficient, and personalized therapeutic strategies. The convergence of these multidisciplinary approaches holds immense potential for developing transformative treatments for neural injuries and neurological disorders, ultimately improving functional recovery.

## Background

Neural regeneration has emerged as a pivotal research direction in neuroscience, aiming to address nerve dysfunction caused by injury or disease. The mammalian nervous system, especially the central nervous system (CNS), exhibits extremely limited regenerative capacity (He and Jin 2016; Varadarajan et al. 2022), rendering injuries or degenerative conditions such as spinal cord injury (SCI) (McKerracher 2001; Tomé and Almeida 2024), stroke (Rust et al. 2024), Alzheimer's disease (AD) (McDowall et al. 2024), and Parkinson's disease (PD) (Huenchuguala and Segura-Aguilar 2024) particularly devastating. Nevertheless, recent innovations in genetic engineering (Lindborg et al. 2021; Liu et al. 2018), materials science (Evans et al. 2024; Li and He 2024; Liu et al. 2024b), and imaging (Deng et al. 2024; Schaffran et al. 2019; Wu et al. 2024) have opened new avenues for promoting neural regeneration. Stem cell-based approaches, including the use of induced pluripotent stem cells (iPSCs) and neural progenitor cells, have demonstrated significant potential in modeling neurological disorders, replacing lost neurons, and promoting synaptic reconnection (Strnadel et al. 2018; Tigner et al. 2024; Wertheim et al. 2022). Additionally, gene editing tools are being harnessed to modulate gene expression and enhance the regenerative potential of neurons. Biomaterials, such as hydrogels and nanofibers, are also being engineered to create scaffolds that maintain neuronal survival, guide axonal growth, and support neural network reconstruction in basic, translational, and clinical research. Furthermore, optogenetics and electrical stimulation techniques are being explored to control neural activity and promote functional recovery precisely (Eldar et al. 2026; Jung et al. 2019; Romeni et al. 2025; Zhou et al. 2024). These technologies are paving the way for transformative treatments for conditions, such as SCI, optic nerve injury, and neurodegenerative diseases.

In recent years, integrating multidisciplinary approaches has become increasingly prominent, particularly with the convergence of neuroscience and cutting-edge technologies. Significant advances across various technological areas, ranging from molecular and cellular levels to systemic and behavioral levels, have unlocked previously inaccessible scientific issues, propelling the field of neuroscience forward. The coordination of these technologies has enabled more comprehensive and in-depth research in neuroscience, significantly advancing our understanding of neurobiology and neural regeneration. In this review, we focus on the application and progress of the latest cutting-edge technologies in neural regeneration research across various scales, highlighting their potential to revolutionize the field.

## Emerging molecular technologies in neural regeneration

### Optogenetics

**Fig. 1:**
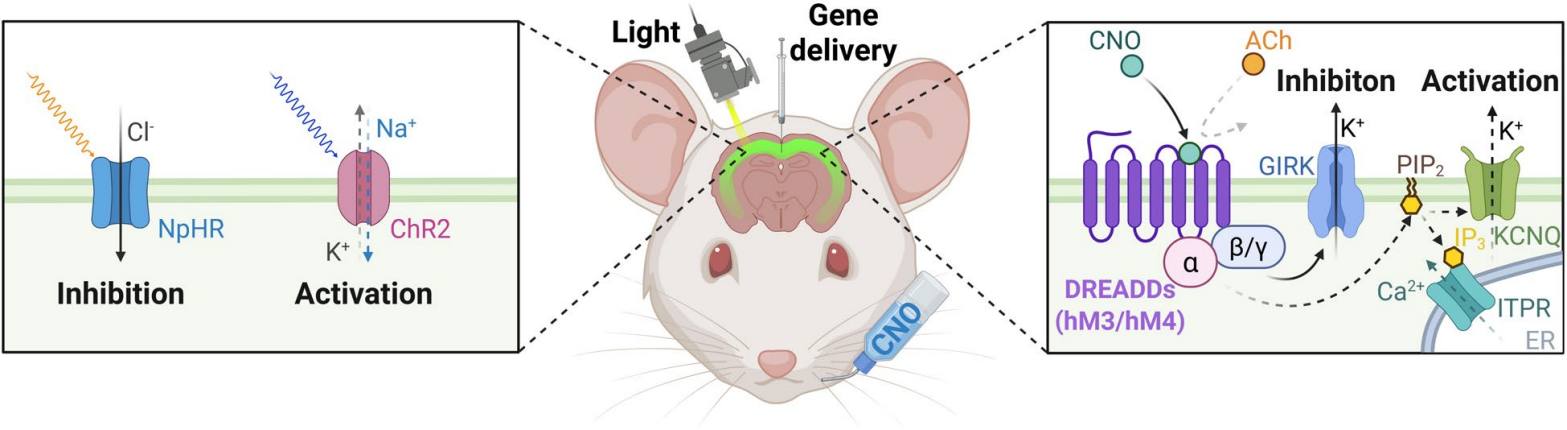
Principles of optogenetics (left) and chemogenetics (right). The application of optogenetic and chemogenetic tools allows for precise manipulation of neuronal functions, facilitating the study of molecular mechanisms and strategies for axonal regeneration. Created with BioRender

Optogenetics is an innovative technology that combines principles of optics and genetics to achieve precise control over the gain or loss of function in specific cells or tissues using light (Fig. 1). The technology operates through a series of steps: first, engineered genes are delivered to target cells or tissues to ensure sufficient expression levels; second, specific neuronal activities are triggered by delivering light stimuli in vitro or in vivo; and finally, the optogenetic modulation of cellular or tissue functions is observed and measured (Manoilov et al. 2021). With continuous advancements and refinements, optogenetics has evolved to manipulate the function and localization of various proteins and signaling pathways involved in critical cellular processes, enabling rapid correlations of biological phenomena across different levels (Emiliani et al. 2022). The nervous system, composed of billions of interconnected cells, exhibits remarkable spatial and temporal precision, making precise regulation essential for its proper functioning. Optogenetics, with its ability to control individual neurons with millisecond precision, is poised to revolutionize neuroscience research.

As a powerful tool in neuroscience, optogenetics enables precise modulation of specific behaviors by inducing gain or loss of function in targeted neurons (Tye and Deisseroth 2012). Its widespread adoption has significantly enhanced our understanding of neural circuits, physiological processes, and the mechanisms underlying neurological disorders, while also accelerating the discovery of novel therapeutic targets (Rost et al. 2022; Tye and Deisseroth 2012). Although the application of optogenetics in neural regeneration is still in its infancy, it holds immense promise. If challenges such as efficient gene delivery, fiber-optic implantation, and minimizing photothermal effects can be addressed in the future, optogenetics could become an invaluable tool for basic research in neural regeneration (Eldar et al. 2026). Optogenetic stimulation has been utilized to examine the functional integration of regenerated corticospinal tract (CST) axons in SCI models (Dias et al. 2018). Furthermore, optogenetics provides crucial validation of synaptic formation by regenerated CST axons, precisely monitoring and optimizing functional reconnection by newly sprouted axons in the injured CNS (Jayaprakash et al. 2016). In addition, optogenetics has been employed to selectively stimulate channelrhodopsin 2-expressing neurons, revealing that targeted neuronal stimulations in the primary motor cortex facilitate functional recovery (Cheng et al. 2014).

Moreover, it has the potential to be developed into a clinical treatment for neural injuries, offering a therapeutic alternative with fewer side effects. Potential applications of optogenetics in neural regeneration include increasing intracellular calcium levels to activate intrinsic regenerative programs, enhancing the plasticity of healthy axons, promoting axonal myelination, and restoring normal neural circuits. These capabilities highlight the transformative potential of optogenetics in advancing both research and clinical interventions in neural regeneration.

### Chemogenetics

Chemogenetics, also known as pharmacogenetic technology, has emerged as a groundbreaking innovation alongside optogenetic technology in recent years (Atasoy and Sternson 2018; Mirabella and Fenselau 2023; Roth 2016). This cutting-edge approach utilizes advanced bioengineering techniques to modify specific proteins, enabling them to interact with previously unrecognized small chemical molecules (Atasoy and Sternson 2018). Through precise modulation of these molecules, chemogenetic technology facilitates controllable and reversible regulation of protein activity, thereby allowing accurate manipulation of protein functions and signaling pathways (Fig. 1). Current chemogenetic tools encompass modified ligand-gated ion channels, protein kinases, and metabolic enzymes, with the most widely utilized being engineered G protein-coupled receptors (GPCRs) that are specifically activated or inhibited by designer drugs (Designer Receptors Exclusively Activated by Designer Drugs, DREADDs) (Wang et al. 2021). Given the extensive development of GPCR-targeting drugs, chemogenetic tools like DREADDs are positioned to play a crucial role in advancing drug development and novel intervention strategies for neurological diseases (Clark et al. 2024; Song et al. 2022). Chemogenetics offers targeted, noninvasive regulation of neuronal activity, which is instrumental in elucidating neural circuit states during development, injury, or disease and establishing causal relationships between these circuits and behavior (Mirabella and Fenselau 2023; Roth 2016). Compared to optogenetic technology, chemogenetic methods are simpler, safer, and capable of sustaining neuronal activation or silencing for extended periods (up to several hours) without disrupting normal cellular physiology. Its applications span diverse research areas, including signaling pathway investigations, functional regulation studies, and neurological disease-related drug development (Neřoldová and Stuchlík 2024; Pereira et al. 2023).

The emergence of chemogenetic technology has revolutionized various neuroscience research domains, particularly in the field of neural regeneration. The capacity to modulate neuronal activity, whether acutely or chronically, has proven invaluable in advancing our understanding of nerve injury responses, axonal regeneration, and circuit reconstruction. For example, chemogenetic stimulation using DREADDs in conjunction with clozapine has been shown to effectively promote functional regeneration following SCI, which results from the modulation of neuroplasticity (Kim et al. 2025). Moreover, combinatorial strategy pairing *RhoA/Pten* deletion with chemogenetic stimulation in corticospinal neurons enables greater axonal regeneration, presynaptic formation, and motor recovery following SCI, compared to *RhoA/Pten* deletion alone (Takatani et al. 2025). Beyond SCI, chemogenetic strategies also advance optic nerve regeneration. Increasing neural activity mediated by chemogenetics promotes long-distance, targeted-specific regeneration of retinal ganglion cell (RGC) axons, thereby leading to partial visual recovery (Lim et al. 2016). Interestingly, chemogenetic inhibition of neuronal activity in the primary motor cortex of ischemic stroke mice improves neuronal survival (Wang et al. 2020) and alleviates neuroinflammation (Li et al. 2025; Xin et al. 2024), supporting that targeting neural activity is a promising strategy in stroke therapy.

In addition to neuronal applications, chemogenetic modulation has been successfully extended to other cell types involved in neural repair processes, such as microglia. For instance, chemogenetics-based exogenous activation of microglial Gi signaling transiently inhibits microglial process dynamics, reduces neuronal activity, and impairs neuronal synchronization, eventually regulating neuronal circuit function for brain homeostasis (Zhao et al. 2025). Additionally, chemogenetic activation of microglia reverses sex-specific deficits in synaptic functions exposed to early-life adversity through restoring normal phagocytic activity (Ahmed et al. 2024). This expansion facilitates a more comprehensive understanding of molecular and cellular responses to neurological disorders, with particular emphasis on the roles of non-neuronal cells in these processes. Taken together, the implementation of chemogenetic technologies promises to significantly enhance our understanding of the mechanisms underlying nerve injury and regeneration. This advancement is expected to catalyze the development of innovative therapeutic strategies and holds substantial potential for applications across basic, translational, and clinical research domains in the foreseeable future.

Optogenetics and chemogenetics represent two powerful neuromodulation approaches with distinct characteristics in spatiotemporal precision, invasiveness, and temporal dynamics, which determine their respective applications in neuroscience research and potential clinical translation. Optogenetics offers millisecond-scale temporal precision and cellular or subcellular spatial resolution through targeted light delivery, enabling precise manipulation of specific neural populations during rapid behavioral processes such as fear conditioning extinction or gamma oscillations (Deisseroth 2015). However, this technique typically requires invasive intracranial hardware (e.g., optical fibers or implanted LEDs) for light delivery, which may cause tissue damage and pose challenges for long-term chronic studies. Moreover, optogenetic effects are transient, ceasing immediately upon light termination. In contrast, chemogenetics operates on slower timescales (minutes to hours) with anatomical rather than cellular resolution, as systemically administered ligands diffusibly activate engineered receptors across entire brain regions (Roth 2016). This approach is minimally invasive, requiring only initial viral vector delivery, and is thus well-suited for longitudinal studies in disease models over weeks or months. Chemogenetic effects last hours to days post-administration, making it particularly valuable for prolonged interventions, such as seizure suppression or Parkinsonian gait improvement (Cachope et al. 2012). In summary, optogenetics is the method of choice for dissecting fast, temporally precise neural dynamics requiring on-demand control, while chemogenetics is better suited for studies or therapeutic applications demanding sustained neuromodulation with reduced implant burden (Raper et al. 2019). The complementary strengths of these techniques enable researchers to address diverse questions in neural circuit function and dysfunction.

## Current cellular technology trends in neural regeneration

### Three-dimensional (3D) cell culture

To elucidate axonal growth mechanisms and characterize growth cone dynamics, researchers have developed a diverse array of in vitro axonal growth models utilizing primary neurons from various species, including Aplysia californica neurons (Forscher et al. 1987; Schacher and Proshansky 1983), chicken dorsal root ganglion (DRG) neurons (Fang et al. 2012; Levi and Lattes 1968), rodent DRG neurons (Hollowell et al. 1990), and hippocampal/cortical neurons (Azmitia et al. 1978; Higuchi et al. 2024; Sekine et al. 2018), among others (Moore et al. 2009). Traditionally, primary neuron cultures have predominantly employed two-dimensional (2D) culture systems. Although widely used in axonal growth and regeneration research, 2D culture systems lack the appropriate environmental context and structural architecture found in vivo, leading to altered cell functions (Lei et al. 2024). To address this Limitation and better replicate physiological conditions, 3D neuronal culture has emerged as a promising alternative. By more accurately recapitulating critical cell-extracellular matrix interactions, 3D culture systems preserve essential cellular characteristics observed in native tissues, including morphology, polarity, cellular activity, and cellular heterogeneity (Ernst et al. 2009; Esposito et al. 2024; Ghosh et al. 2005; Weaver et al. 1997). For instance, the growth cone cytoskeleton exhibits distinct structural organization and dynamics in 3D environments compared to 2D systems, resulting in marked differences in axonal growth cone morphology. Furthermore, axonal elongation in 3D cultures is not only faster but also more dynamic and physiologically relevant than in traditional 2D cultures (Santos et al. 2020).

**Fig. 2:**
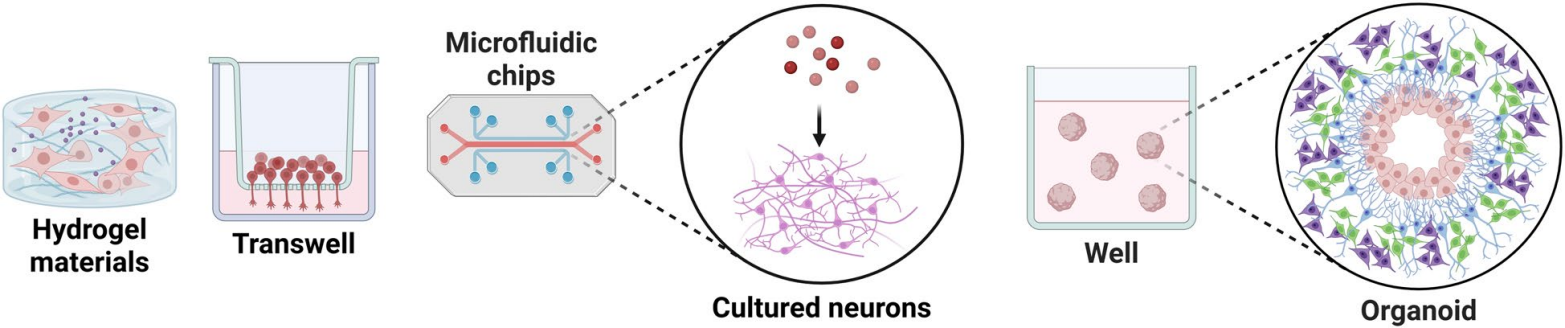
3D culture of neurons. The use of microphysiological systems and biomaterials facilitates the 3D culture of neuronal cells, organoids, or tissues, and carries out modeling of approximate physiological states, mechanism research, and drug screening. For example, the development of hydrogel materials, microfluidic chips, and organoid technology provides solutions for constructing a similar in vivo microenvironmental neuronal culture system. Created with BioRender

Certainly, it is important to recognize that current 3D neuronal culture systems are not without limitations. The simplified matrix environment of these systems constrains their ability to fully replicate the intricate complexity of nervous tissue (Fig. 2). The fusion of microphysiological systems and advanced biomaterials (such as hydrogels and nanofibers) has enabled the encapsulation of neural cells and tissues within 3D environments, allowing for the controlled study of various biological processes (Gabriel et al. 2023; Linsley et al. 2024). For example, responsive hydrogels containing magnetic nanoparticles enable dynamic remodeling of extracellular microenvironments that precisely mimic the mechanical properties of natural tissues for neuronal viability and axonal growth (Li and He 2024; Martinez-Ramirez et al. 2024; Tran et al. 2022). Concurrently, conductive scaffolds composed of conductive components not only provide mechanical support but also enable electrical stimulation (ES) that mimics natural neural signal transmission, thereby promoting neuronal differentiation and enhancing neurite outgrowth (Li and He 2024; Murphy et al. 2017). These 3D culture systems not only facilitate the study of axonal growth and neuroprotection but also support the development of sophisticated in vitro models of neural diseases and injuries (Broguiere et al. 2019; Meng et al. 2023). Recent studies have shown that human neural stem cells (hNSCs), integrated into 3D cell- or non-cell-autonomous culture systems for AD modeling, develop key representative features of AD neuropathology, including beta-amyloid aggregation, phosphorylated tau accumulation, and neuroinflammatory activity (Hebisch et al. 2023; Kwak et al. 2020; Papadimitriou et al. 2018; Park et al. 2018). Inspiringly, using 3D bioprinting technology and biomaterials, 3D-loaded stem cell therapy for SCI serves as a potential strategy that generates spatial pore architecture and mechanical characteristics to promote neural regeneration, thereby resulting in functional recovery (Jiu et al. 2025). In addition, embedding ex vivo nervous tissue within a 3D hydrogel environment is utilized to explore the process of neural regeneration and pharmacodynamic evaluation (Zhang et al. 2023). Collectively, these innovations in biomaterial design and 3D culture technology have established next-generation platforms that are revolutionizing both mechanistic studies and therapeutic development in regenerative neuroscience.

### Cellular microarray

Cellular microarrays, commonly referred to as cell-on-a-chip or microfluidic systems, involve the cultivation of living cells within microengineered platforms (Berthuy et al. 2016; Rothbauer and Ertl 2018). This innovative technology enables the creation of in vitro cellular models that more accurately mimic the physiological conditions, thereby yielding more reliable and physiologically relevant results compared to traditional cellular models. In recent years, cell-on-a-chip technology has demonstrated significant potential in advancing molecular and cellular biology research (Gupta et al. 2019; Tsougeni et al. 2018). Notably, microfluidic systems leveraging 3D printing technology have revolutionized the spatiotemporal regulation of cell growth and environmental stimuli in vitro, offering unparalleled advantages such as miniaturization, integration, automation, and customization (Fig. 2).

Microfluidic devices have revolutionized neurobiological research by providing unprecedented capabilities that surpass conventional methodologies (Kang et al. 2023; Wan et al. 2022). These innovative platforms offer precise spatiotemporal control over neuronal microenvironments, enabling targeted delivery of biochemical and physical stimuli at both network and single-cell levels. A key advantage Lies in their ability to maintain highly stable culture conditions through continuous real-time monitoring systems. The merging of advanced 3D microarchitectures further enhances their utility by facilitating high-throughput multi-omics analyses through efficient biomolecular and cellular sorting (Wang et al. 2024). Moreover, these devices provide researchers with exceptional experimental control, allowing systematic investigation of neuronal morphology, functionality, and behavior through precise manipulation of critical parameters, including hydrodynamic flow characteristics, microchannel geometry, and substrate material properties. Thus, the technological versatility of these devices has opened new avenues for investigating fundamental neurobiological processes, such as ion channel function, intercellular communication, NSC differentiation, axonogenesis, and myelination. For instance, the merging of embedded microgrooves within these devices allows neuronal somata to be confined to specific compartments while enabling axons to extend through the microgrooves to adjacent compartments. This configuration of the devices creates a highly adaptable system, ideal for in vitro studies of neurodegeneration, axon injury, and axonal growth. Therefore, microfluidic devices are particularly advantageous for investigating the mechanisms underlying neural regeneration, exploring the pathological changes of neurodegenerative diseases, and developing high-throughput drug screening (Feng et al. 2024). Beyond the aforementioned applications in AD modeling, microfluidic platforms have also proven invaluable for investigating the regulatory mechanisms of axonal regeneration in amyotrophic lateral sclerosis (ALS) models (Marshall et al. 2023).

Despite these remarkable advancements, the design of microfluidic devices remains in its early stages, with most devices currently used in neuroscience research being relatively simplistic. However, the future of cell-on-a-chip technology holds immense promise, particularly with the development of cellular microarrays featuring integrated multifunctional modules and automated high-throughput capabilities. While challenges persist in applying this technology to neural regeneration research, ongoing innovations are expected to overcome these limitations, paving the way for more sophisticated and effective tools in the field.

### Organoid

Organoids, self-organized 3D models derived from pluripotent, embryonic, or adult stem cells, replicate the functional, structural, and biological complexity of organs by generating diverse cell types and tissue properties (Corrò et al. 2020) (Fig. 2). In recent years, the use of tissue-resident or iPSC-derived organoids has become increasingly prevalent for modeling the development, function, and pathology of the nervous system in vitro. Neural organoids, generated through 3D cell culture systems, encompass a variety of neural cells capable of mimicking neurodevelopmental processes and exhibiting neurophysiopathological phenotypes. This capability offers significant advantages in exploring regenerative mechanisms and drug discovery. In particular, neural organoids derived from human iPSCs have facilitated the in vitro modeling of human neurodevelopment and the exploration of genetic, molecular, and cellular underpinnings of neurological disorders (Yang et al. 2025a). Moreover, they hold promise as sources for cellular replacement therapies and as platforms for therapeutic strategy screening and evaluation in human neural cells.

With prolonged in vitro culture, iPSC-derived neural organoids have been observed to reach millimeter dimensions and manifest microscale structures, cellular phenotypes, and neural circuits, analogous to those of developing neural tissues. Current methodologies primarily involve inducing the spontaneous differentiation of stem cells into neural organoids that reflect region-specific neural structures. Additionally, the use of small molecules or recombinant proteins to mimic developmental signals and guide differentiation into specific neural structures is advancing. This approach has yielded neural organoids with complex architectures, including those resembling the dorsal forebrain, ventral forebrain, thalamus, hypothalamus, hippocampus, cerebellum, choroid plexus, and retina. These models enable the exploration of human neurodevelopmental, physiological, and pathological traits at multiple levels, enhancing our understanding of neural function in health and disease. Neural organoids have also emerged as indispensable tools for elucidating the molecular mechanisms of neural regeneration. In a recent study, cortical organoids derived from Richieri-Costa-Pereira syndrome patient harboring loss of function mutations in the *EIF4A3* gene exhibit significant impairments in neuronal growth, providing compelling evidence for its conserved roles in neurodevelopmental pathology (Alsina et al. 2024). Furthermore, groundbreaking research using human iPSC-derived myelin organoids has revealed that small molecule-induced epigenetic rejuvenation can effectively promote CNS myelin regeneration, highlighting a potential strategy for remyelination in various neurodegenerative diseases and aging-related cognitive decline (Liu et al. 2024a).

Recent studies have been devoted to evaluating the strengths and limitations of neural organoids using different methodologies and refining culture methods to facilitate their expansion. Despite their utility, neural organoids are inherently artificial and simplistic compared to the intact nervous system. Future research will focus on refining culture techniques and integrating bioengineering technologies such as biomaterials, gene editing, and microfluidics to enhance the spatiotemporal control of organoid development. These advancements are expected to improve the structural and functional fidelity of organoids, making them more suitable for studying disease mechanisms, developing regenerative therapies, and screening drugs. Consequently, neural organoids are poised to revolutionize neural regeneration research and therapeutic strategies for intractable CNS injuries and neurodegenerative disorders.

## Innovative technologies and methods in neural regeneration

### Gene editing

Since the identification of genes as the basic units of hereditary information responsible for the regulation of biological characteristics, the field of biomedicine has sought to achieve fundamental cures for diseases through genetic modification. Gene editing, a revolutionary biotechnology, has been demonstrated to efficiently and precisely insert, delete, or replace specific target genes (Doudna and Charpentier 2014), enabling targeted gene enhancement, inhibition, and editing, thereby altering genetic information and phenotypic characteristics. The foundation of gene editing lies in DNA double-stranded breaks (DSBs), which activate the endogenous DNA repair mechanism (Jasin and Rothstein 2013). When a site-specific DSB is introduced at a specific genomic location, the broken DNA is repaired through homologous recombination (HR) and non-homologous end joining (NHEJ), thus completing the gene editing process (Lieber 2010; Roth 2014) .

**Fig. 3:**
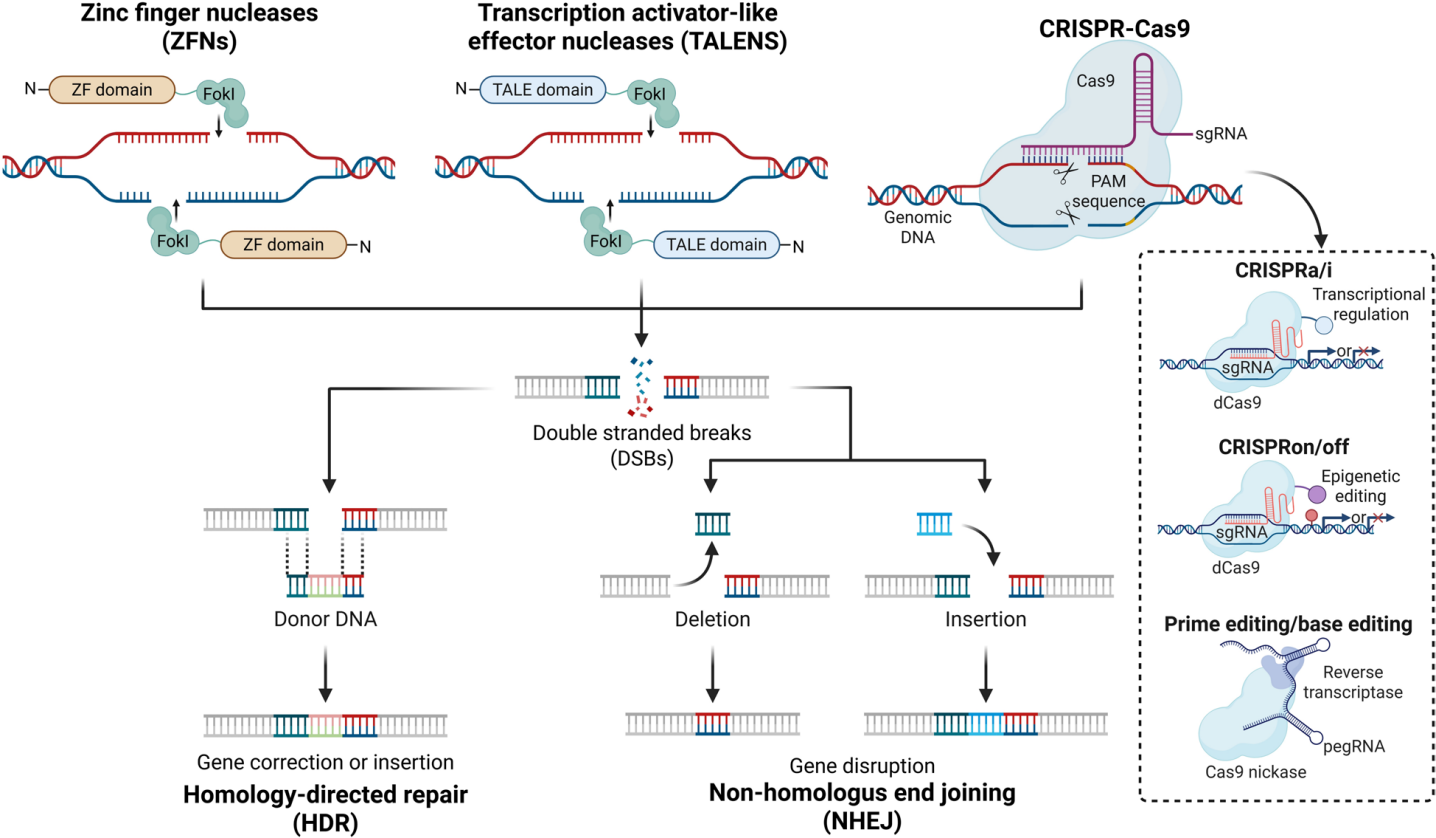
Principles of gene editing technology. Gene editing technology utilizes sequence-specific nucleases to recognize specific DNA target sequences to create double-strand breaks (DSBs) and then repair the broken DNA by homologous recombination (HR) and nonhomologous end joining (NHEJ) to enable specific genetic modification. The initial generation of gene editing instruments, zinc finger nucleases (ZFNs), fuses the non-specific cleavage domain of FokI endonuclease with a zinc finger domain, offering a general way to deliver a site-specific DSB to the genome. The second-generation tool, transcription activator-like effector nucleases (TALENs), consists of a nonspecific DNA-cleaving FokI nuclease domain fused to a customizable DNA-binding domain (TALE) to introduce targeted DSBs. The CRISPR-Cas9 system, known as the third-generation gene editing technology, involves an endonuclease guided by a single guide RNA (sgRNA), which binds to the target DNA site of the protospacer adjacent motif (PAM) and produces DSBs for targeted genome modification. Recently, a variety of novel editing tools have been developed based on the CRISPR strategy, including CRISPRi and CRISPRa for gene suppression and activation, CRISPRoff and CRISPRon for epigenetic editing, base editors, and prime editors for long fragment editing. Created with BioRender

To date, the most widely used gene editing technologies include zinc finger nucleases (ZFNs), transcription activator-like effector nucleases (TALENs), and clustered regularly interspaced short palindromic repeats (CRISPR) (Fig. 3) (Doudna and Charpentier 2014; Joung and D. Sander 2013; Urnov et al. 2010). The development of precise DNA-binding modules and the FokI restriction endonuclease has enabled the utilization of zinc finger proteins and transcription activator-like effectors in ZFNs and TALENs, respectively, for genome editing (Miller et al. 2011). Shortly after the discovery of ZFNs and TALENs, the CRISPR-CRISPR associated protein (Cas)9 system (CRISPR-Cas9) emerged as a third-generation gene editing technology. Due to its high efficiency, low cost, and ease of use, CRISPR-Cas9 has become the dominant tool of genetic modification (Hsu et al. 2014). The principle of CRISPR-Cas9-mediated genome editing is straightforward: the Cas9 protein cuts the target DNA sequence, creating a DSB (Cong et al. 2013). The DNA repair system then repairs the break, often resulting in a change in the DNA sequence during the repair process. In recent years, the field of gene editing has advanced rapidly, with significant progress in the development and application of CRISPR-based tools (Kleinstiver et al. 2016). The identification of multiple Cas homologs and variants has expanded the target range and increased editing specificity, as evidenced by the emergence of Cas9n, dCas9, Cas12, and Cas13 (Abudayyeh et al. 2016; Chen et al. 2018; Qi et al. 2013). Beyond the well-established HR and NHEJ strategies, novel CRISPR-based approaches have been developed, including CRISPRi and CRISPRa for gene silencing and activation (Gilbert et al. 2014), CRISPRoff and CRISPRon for epigenetic editing, RNA aptamers for recruiting RNA-binding proteins (Nuñez et al. 2021), base editors for single base pair editing (Komor et al. 2016), and primer editors for long fragment editing (Fig. 3) (Anzalone et al. 2019).

Since its introduction, gene editing technology has become a widely adopted research tool in the life sciences, enabling the rapid generation of cellular and animal models, functional genomic screens, and live imaging of the cellular genome, among other applications. Moreover, gene therapy based on gene editing has emerged and is gradually expanding its potential clinical applications in various diseases, including genetic disorders, infectious diseases, and cancers. In the field of neural regeneration, the CRISPR-Cas system has been instrumental in precisely regulating critical molecules, elucidating molecular mechanisms, and identifying therapeutic targets (Hsu et al. 2014; Konermann et al. 2015; Swiech et al. 2015). Additionally, the development of novel CRISPR-Cas gene editing tools has shown significant potential for application in gene therapy for neurological injuries, disorders, and diseases. Human embryonic stem cell-derived oligodendrocyte progenitor cells (OPCs) with CRISPR-Cas9-mediated *NRP1* deletion are transplanted into rodent models of demyelination lesions, which overcomes the inhibitory microenvironment that restricts remyelination, demonstrating therapeutic potential for progressive multiple sclerosis (MS) (Wagstaff et al. 2024). Notably, several of these tools have already been successfully employed in clinical settings. For instance, gene therapy for ophthalmic diseases has made significant progress, particularly in clinical applications. Specifically, CRISPR-based gene editing strategies have emerged as promising therapeutic approaches for neuroprotection, optic nerve regeneration, and intraocular pressure reduction in glaucoma, holding considerable potential for widespread clinical use (Bei et al. 2016).

Furthermore, the CRISPR-Cas system's ability to generate a large number of mutant cells enables systematic analysis to determine whether observed phenotypic changes are associated with specific mutant genes (Wang et al. 2013). Consequently, CRISPR-based gene screening offers high specificity, high efficiency, and in vivo applicability (Shalem et al. 2015). For example, high-throughput screening of regeneration-associated genes (RAGs) can be conducted using the CRISPR-Cas-mediated gene editing tools. Notably, 40 promising regeneration-limiting genes are screened by the CRISPR-Cas9 system, whose loss of function enhance axonal regeneration in vivo (Lindborg et al. 2021). The CRISPR-Cas system has also been extensively utilized in developing in vitro and in vivo models of nervous system diseases, including neurodevelopmental disorders, neurodegenerative diseases, and genetic conditions (Bilkei-Gorzo 2014; Yang et al. 2017). Moreover, CRISPR gene editing technology enables live-cell genome imaging (Chen et al. 2013), allowing simultaneous regulation and labeling of multiple specific gene loci, thereby facilitating a comprehensive understanding of the dynamics of gene regulatory networks during neurological disease or treatment.

Nevertheless, the implementation of gene editing technology, especially in clinical gene therapy, requires careful assessment of ethical concerns and potential risks, as well as an improved regulatory framework to ensure safety and sustainability. The successful clinical translation of CRISPR-based therapeutics necessitates overcoming two fundamental challenges: minimizing off-target effects and improving delivery efficiency. Recent technological breakthroughs have substantially addressed these limitations through multiple innovative strategies. High-fidelity Cas9 variants (e.g., SpCas9-HF1, eSpCas9, and HypaCas9) have been engineered with enhanced proofreading mechanisms, significantly reducing off-target mutations compared to wild-type Cas9 (Chen et al. 2017; Kleinstiver et al. 2016; Slaymaker et al. 2016). Additionally, DNA break-free editing systems, such as base editor and prime editor, enable precise nucleotide conversion without double-stranded DNA cleavage, thereby mitigating risks of genomic instability (Anzalone et al. 2019; Gaudelli et al. 2017). Delivery challenges, particularly for neurological applications, are being addressed through engineered adeno-associated virus (AAV) capsids, which offer a favorable safety profile and efficient neuronal targeting. These collective advances have propelled CRISPR technology into clinical development, with over 300 completed or ongoing trials, more than a third of which target neurological disorders (Ling et al. 2023). In short, these innovations underscore CRISPR's potential as a robust and precise therapeutic platform for treating neurological diseases.

### Single-cell sequencing

In recent years, single-cell sequencing technology has emerged as a transformative research tool in the life sciences. It has been widely applied across various fields, including developmental biology, disease mechanisms, tumor resistance, and neuroscience (Jovic et al. 2022; Tirosh et al. 2016; Wagner et al. 2018). Due to its unparalleled single-cell resolution and high-throughput capabilities, this technology enables precise analysis of cellular states under diverse pathophysiological conditions. By integrating multi-modal profiling, single-cell sequencing allows for the comprehensive analysis of genomes, transcriptomes, and epigenomes (Buenrostro et al. 2015; Clark et al. 2018; Stuart et al. 2019; Svensson et al. 2018). This approach not only identifies cell types and tracks changes in cellular states but also reveals cellular heterogeneity and uncovers the molecular and cellular mechanisms underlying complex biological phenomena at the single-cell level (Setty et al. 2019; Trapnell et al. 2014).

**Fig. 4:**
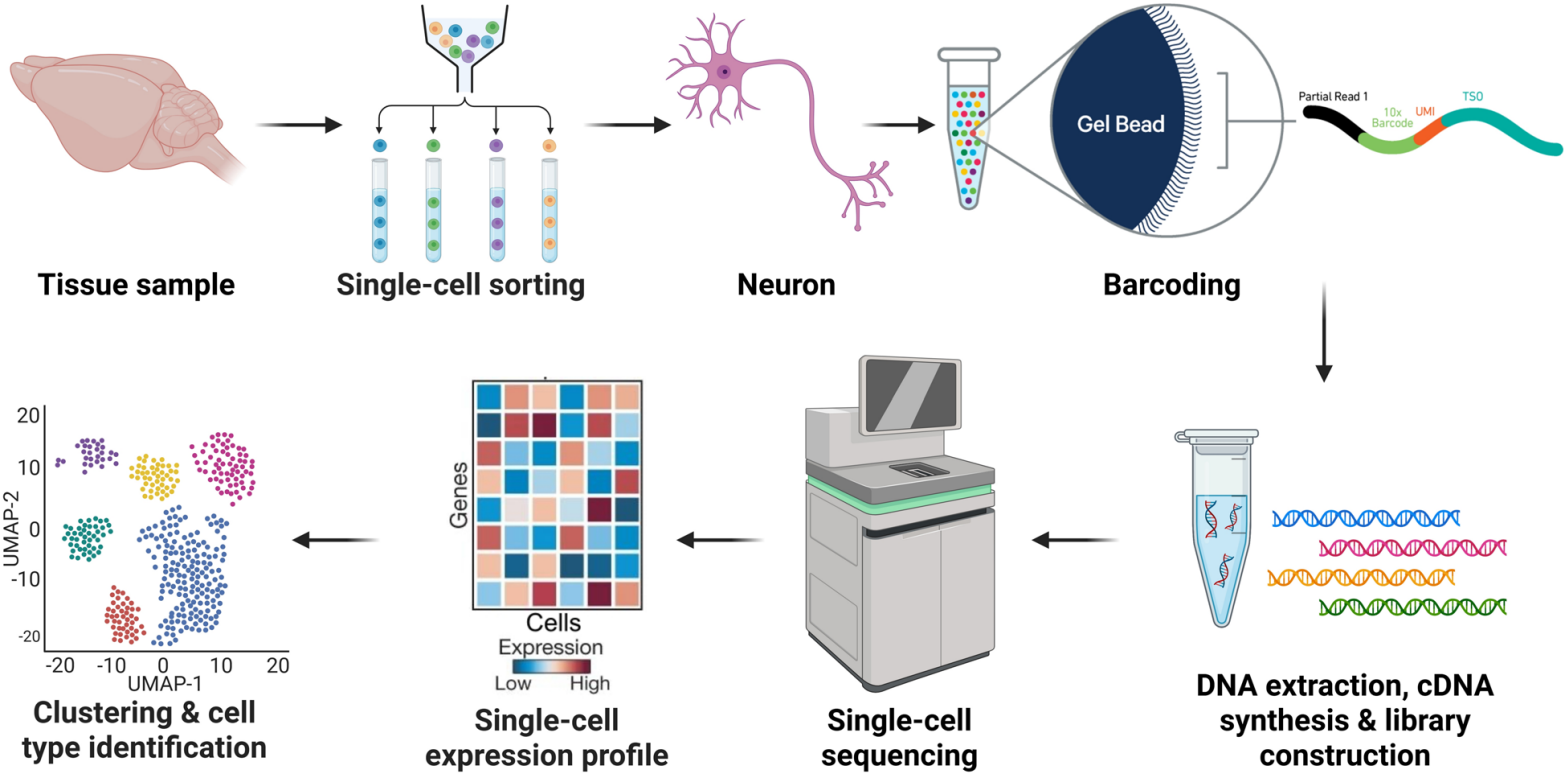
scRNA-seq workflow. The scRNA-seq procedure includes the following steps: single-cell suspension preparation, cell sorting, adding barcodes, library construction, high-throughput sequencing, and data analysis. This workflow enables comprehensive transcriptomic profiling at single-cell resolution, facilitating the investigation of cellular heterogeneity and gene expression dynamics within neural tissues. Created with BioRender

The core principle of single-cell sequencing technology includes three key steps: single-cell isolation, amplification and sequencing, and data analysis (Fig. 4). Since the introduction of the first single-cell transcriptome sequencing (scRNA-seq) technology in 2009 (Tang et al. 2009), this field has rapidly advanced. Innovations in single-cell isolation, nucleic acid amplification, high-throughput sequencing, and data mining have led to the development of a suite of single-cell omics technologies, including scWGS (Buenrostro et al. 2015), scWGBS (Smallwood et al. 2014), scHi-C (Nagano et al. 2013), scChIP-seq (Rotem et al. 2015), and scATAC-seq (Buenrostro et al. 2015). More recently, cutting-edge techniques such as single-cell/nucleus multi-omics sequencing, single-cell spatial omics, as well as deep learning-based cell–cell communication have emerged (Ståhl et al. 2016; Stoeckius et al. 2017). These advancements, along with single-cell proteomics and metabolomics based on mass spectrometry, provide a more comprehensive and refined analytical framework (Budnik et al. 2018; Shrestha 2020). They enable researchers to dissect cellular heterogeneity, map tissue microenvironments, and track pathophysiological processes, offering novel insights for clinical disease prevention, diagnosis, and treatment.

Cellular heterogeneity is a hallmark of multicellular organisms, closely Linked to cellular function and state. The nervous system, with its exceptional structural and functional complexity, exemplifies this heterogeneity. For instance, the mouse retina comprises approximately 130 distinct neuronal types (Shekhar et al. 2016), and similar diversity is observed in the brain. Single-cell sequencing technology has become an invaluable tool for unraveling this complexity, providing multi-dimensional insights into the characteristics of neurons. Similarly, a common feature of nerve injury, aging, and disease is the pronounced heterogeneity in axonal regeneration and survival capacity among neuronal subpopulations. Traditional strategies to identify key molecules regulating neuroprotection and axonal regeneration have relied on comparisons across developmental stages, injury time points, or species (François et al. 2017). Single-cell sequencing, however, offers a powerful alternative by characterizing gene expression profiles across neuronal subclasses before and after injury, revealing heterogeneity in neuroprotection and regeneration, and identifying potential therapeutic targets (Toma et al. 2016). A prime example is the application of scRNA-seq to construct a detailed cellular atlas encompassing 46 distinct subtypes of RGCs (Rheaume et al. 2018; Tran et al. 2019). This comprehensive atlas has enabled researchers to precisely track survival rate variations among all RGC subtypes following axonal injury and to identify key regulatory molecules associated with neuronal survival and axonal regeneration (Li et al. 2022; Tian et al. 2022; Tran et al. 2019).

The complexity of tissue composition, where multiple cell types synergistically interact to maintain physiological functions, is particularly evident in the nervous system. This intricate network comprises not only diverse neuronal populations but also various classes of glial cells, which engage in complex communication through multiple mechanisms. These include direct intercellular interactions via cell junctions, as well as cell–cell communications mediated by ligand-receptor interactions (Xin et al. 2022). The combination of scRNA-seq with spatial transcriptomic technologies has opened new avenues for analyzing cell–cell communications within the nervous system (Moffitt et al. 2018; Ståhl et al. 2016), allowing to elucidate complex regulatory roles and mechanisms of the neural microenvironment in nerve injury, aging-related degeneration, and neurological disorders. In summary, single-cell (multi)omics technologies serve as an indispensable tool for uncovering dynamic changes of neuronal intrinsic regulatory networks and tissue microenvironment in nerve injury and repair processes.

### 3D imaging

Most biological tissues are inherently opaque, making it impossible to directly observe their fine internal structures using conventional microscopy (Hillman et al. 2011). For instance, the high light-scattering properties of tissues like the brain pose significant challenges for deep-tissue imaging (Yuste 2015). Traditional methods, such as fluorescence or electron microscopy of mechanically sectioned tissues, are commonly used to obtain structural and morphological information at cellular and subcellular levels (Denk and Horstmann 2004). However, these approaches have drawbacks such as sample distortion, loss of fine details, low throughput, and high demands on time and labor (Dodt et al. 2007). Consequently, achieving deep-tissue imaging with subcellular resolution has become a critical need in biomedical research, enabling more accurate restoration of cellular morphology and tissue architecture.

**Fig. 5:**
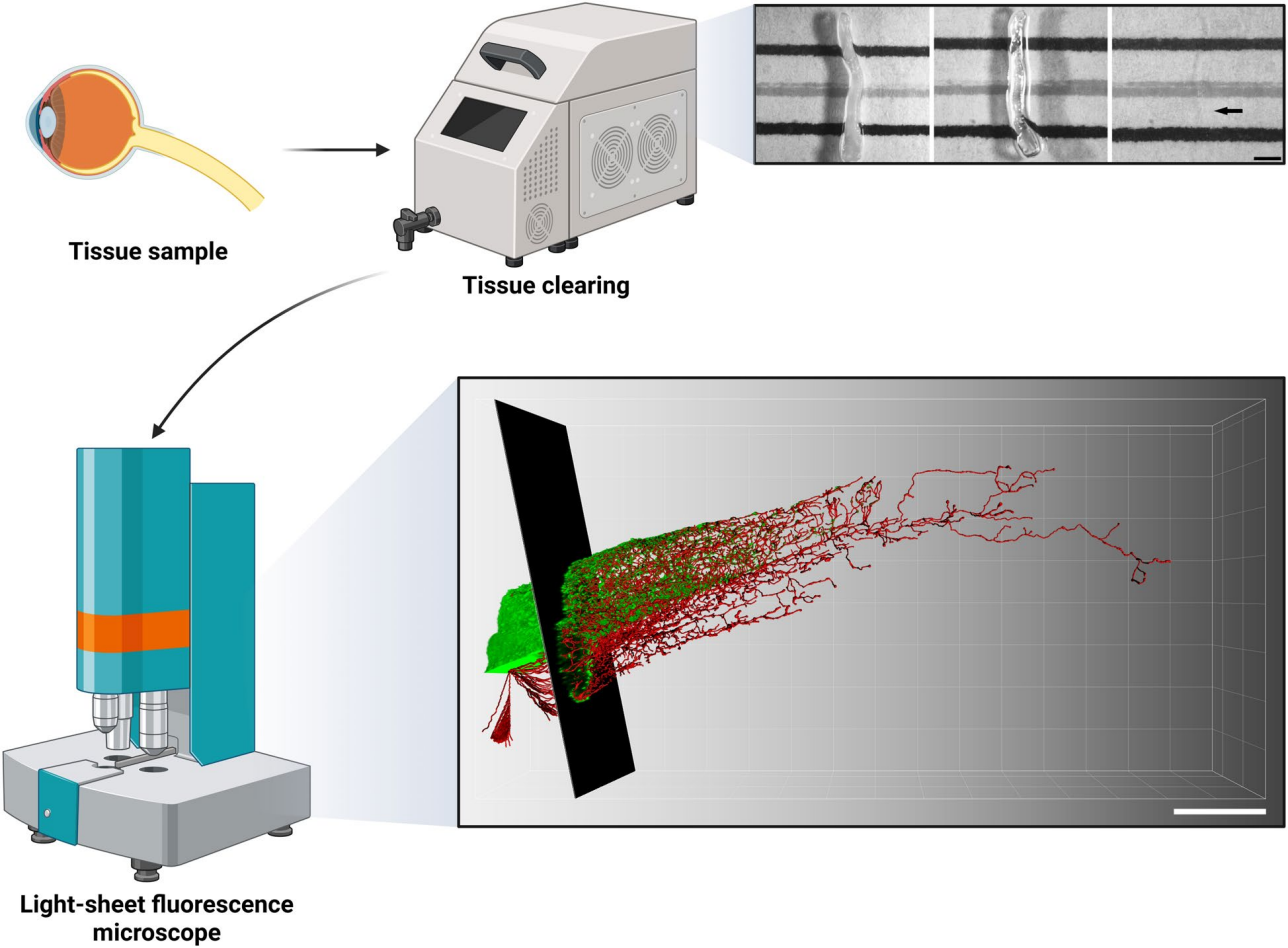
3D imaging of neural tissues using advanced tissue clearing and Light-sheet microscopy. The combination of tissue clearing methods with high-resolution Light-sheet microscopy enables multiscale 3D reconstruction of regenerated axons. This cutting-edge imaging platform offers a powerful tool for investigating structural and functional relationships within complex neural networks at cellular and subcellular resolution. Created with BioRender

Recent advancements in tissue clearing techniques and Light-sheet microscopy have revolutionized the field, making micrometer-scale 3D imaging of whole tissues a preferred method for studying large biological samples (Fig. 5) (Huisken et al. 2004; Ueda et al. 2020). The opacity of biological tissues arises from light scattering caused by variations in the refractive indices of their constituent materials. Tissue clearing methods (Table 1), such as BABB (Dodt et al. 2007), SeeDB (Ke et al. 2013), CLARITY (Chung et al. 2013), DISCO (Ertürk et al. 2012), Sca*l*e (Hama et al. 2011), and CUBIC (Susaki et al. 2014), address this issue by reducing light scattering, thereby enhancing light penetration and improving imaging depth and quality. Complementing these techniques, light-sheet microscopy employs a thin sheet of light to illuminate a single plane of a fluorescent sample (Stelzer 2015). A detector lens, positioned perpendicular to the Light sheet, captures the emitted fluorescence, and by moving the sample through the Light sheet, non-destructive 3D volumetric imaging is achieved (Power and Huisken 2017). Recent innovations have enabled the imaging of cleared and expanded biological tissues with spatial resolutions ranging from micrometers down to 100 nm using tiling light-sheet microscopy (Gao et al. 2012). Additionally, this method allows for rapid acquisition of fluorescent signals, minimizing photobleaching and eliminating out-of-focus background noise (Dean et al. 2015; Tomer et al. 2012).

**Table 1:** Comprehensive methodological comparison of major tissue-clearing techniques

Method	Processing time	Clearing capability	Morphological change	Labeling compatibility	Main chemicals
BABB	3 Days	Strong	Shrinkage	Fluorescent quenching	Benzyl alcohol, Benzyl benzoate
SeeDB	3 Days	Weak	No tissue expansion	No fluorescent quenching	Fructose, α-thioglycerol
CLARITY	2 Weeks	Strong	Tissue expansion	No fluorescent quenching	SDS, Boric acid, FocusClear
DISCO	5~7 Days	Strong	No tissue expansion	No fluorescent quenching within a few days	Dibenzyl ether, Tetrahydrofuran
Sca*l*e	3 Weeks	Medium	Tissue expansion	Partial fluorescent quenching	Urea, Glycerol, Triton X-100
CUBIC	2 Weeks	Strong	Tissue expansion	No fluorescent quenching	Urea, Aminoalcohol, Sucrose, Nitrilotriethanol

The human CNS, composed of trillions of neurons, forms an intricate 3D network that underlies critical functions such as sensory conduction, motor coordination, learning, memory, and cognition (Herculano-Houzel 2009). Understanding the morphological and structural features of this network from a multidimensional perspective is essential for elucidating the state and function of neurons during development, injury, aging, and disease. Studies on neuroprotection and regeneration often require detailed observation and comparison of neuronal and subcellular 3D morphology, as well as neural network architecture, to determine whether regenerated networks can restore normal physiological functions (Economo et al. 2015; Tomer et al. 2014). Traditional methods for analyzing neural regeneration, which rely on tissue sectioning, are Limited by issues such as axonal discontinuity and the loss of 3D spatial information. In contrast, the combination of tissue clearing and Light-sheet microscopy enables 3D imaging of neural tissues, capturing spatial features from the cellular to synaptic levels, including neuronal distribution, dendritic morphology, axon projections, and synaptic connectivity (Eberle et al. 2015; Huisken et al. 2004). This approach provides insights into the dynamic changes in neuronal states and cell–cell interactions during nerve injury and repair, revealing the underlying molecular and cellular mechanisms from a 3D morphological perspective (Kasthuri et al. 2015; Renier et al. 2016). Remarkably, a recent study reveals that the combination of tissue clearing, tissue expansion, and tiling light-sheet microscope enables decoding large neural networks with spatial resolution from micron to sub-100 nm level (Xie et al. 2023).

Despite its advantages, 3D imaging faces challenges such as antibody labeling efficiency, imaging resolution, and the storage and processing of large datasets (Chung et al. 2013). Nevertheless, great progress in 3D imaging technology offers unparalleled flexibility, accuracy, and speed, making it a powerful tool for advancing our understanding of the structure–function relationships in neural tissues (Chen et al. 2016; Power and Huisken 2017). While Light-sheet microscopy represents a powerful approach, alternative 3D imaging technologies, including confocal microscope (Yang et al. 2025b), two-photon/multi-photon microscopy (Kugler et al. 2022), micro-optical sectioning tomography (Zheng et al. 2019), optical coherence tomography (Deloria et al. 2021), and 3D mesoscopy (Zhang et al. 2024), also provide valuable capabilities for morphological features of the nervous system across multiple spatial and temporal scales. In addition, emerging imaging modalities, such as mass spectrometry (Passarelli et al. 2017), magnetic resonance (Lerch et al. 2017), and acoustic-based techniques (Upputuri and Pramanik 2020), have advanced rapidly, enabling 3D visualization of biomolecules at multiple resolutions. Taken together, 3D imaging holds great promise for accelerating discoveries in neurobiology and exploring strategies for neuroprotection and regeneration.

## New perspectives on cutting-edge technologies for neural regeneration

The convergence of multidisciplinary technologies represents a transformative paradigm in the evolution of cutting-edge technologies for biomedical research. In recent years, machine learning (ML) or artificial intelligence (AI)-based single-cell multi-omics technologies have brought revolutionary breakthroughs to the field of neural regeneration. Single-cell multi-omics enables the analysis of genomic, transcriptomic, epigenomic, and proteomic information at the single-cell level (Song and Liu 2022), providing unprecedented resolution for understanding cellular heterogeneity, developmental trajectories, and regenerative mechanisms in neural tissues (Trapnell 2015; Wagner et al. 2018). However, the massive datasets generated by single-cell multi-omics pose significant challenges for traditional analytical methods. The incorporation of ML and AI technologies has made it possible to extract critical biological insights from these complex datasets. For instance, deep learning algorithms can accurately identify key genes and signaling pathways associated with neural regeneration. Recent progress has shown that combining deep scRNA-seq sequencing with ML algorithms is utilized to generate an applicable regeneration classifier, which exhibits strong predictive power for the regenerative potential across neuronal types, anatomical regions, and developmental timeline (Kim et al. 2023). Furthermore, exploiting the heterogeneity of regenerative capacities across neuronal subtypes, bulk RNA-seq-based differential gene expression analysis has identified new regeneration regulators, one of which has been successfully validated by genetic modulation with an in vivo injury model (Kim et al. 2023). Recently, ML-based multi-omics integration methods, such as generative adversarial networks (GANs) and feedforward neural networks (FNNs), enable the generation of high-fidelity synthetic datasets to effectively address data scarcity challenges while extending beyond the traditional molecular omics to incorporate multimodal biological data (Ballard et al. 2024). For structural analysis, convolutional neural networks (CNNs) have achieved automated, high-throughput 3D axon reconstruction from whole-brain optical microscopy images with sub-micron precision (Urakubo 2025). Critically, the rapid development of ML-based methodologies, coupled with the increasing availability of large-scale datasets (e.g., multi-batch and cross-species validation), has significantly reduced data bias, facilitating a more comprehensive understanding of complex biological processes. Additionally, AI-driven models can simulate tissue microenvironmental changes following neural injury, offering new opportunities for understanding pathological processes (Schreiber et al. 2020). The synergy of these technologies not only accelerates the deciphering of neural regeneration mechanisms but also provides powerful tools for developing novel therapeutic strategies, such as stem cell-based neural repair and gene therapies, thereby bridging the gap between basic research and clinical applications. Together, as data integration capabilities and algorithmic performance continue to advance, the fusion of single-cell multi-omics with AI is poised to play an even more pivotal role in advancing neural regeneration research.

In addition, CRISPR gene editing technology offers a potential tool for high-throughput screening of genes related to neural regeneration in vivo, promising to drive significant advancements in the field. In the future, in vivo high-throughput CRISPR screening platforms (Ruetz et al. 2024; Wertz and Heiman 2017) will enable systematic inhibition, activation, or modification of a large number of genes in live animal models, allowing for the rapid identification of key genes and signaling pathways involved in neuroprotection, axonal growth, and synaptic plasticity. By integrating single-cell sequencing and spatial transcriptomics (Ståhl et al. 2016; Zheng et al. 2024), researchers can analyze the effects of gene editing on neuronal behavior, microenvironment remodeling, and functional recovery of neural networks at single-cell resolution. A breakthrough AAV-based Perturb-seq platform has recently been developed, enabling massively parallel genetic perturbation screening across diverse neural tissues and cell types (Zheng et al. 2025, 2024). This innovative system combines high-throughput in vivo gene manipulation with single-cell transcriptomic profiling, allowing comprehensive gene-expression-based characterization at single-cell resolution. Additionally, in vivo CRISPR screening can be combined with ML algorithms to mine potential gene targets and therapeutic strategies from vast datasets (Eraslan et al. 2019), providing insights and support for the development of novel neural regeneration therapies. With the improvements in delivery systems and editing efficiency (Raguram et al. 2022; van Haasteren et al. 2020), the application of in vivo CRISPR screening will become more widespread and efficient, bringing great progress to the field of neural regeneration and accelerating the translation of basic research into clinical treatments.

Another transformative development is brain-computer interface (BCI) technology (Lebedev and Nicolelis 2006), which holds immense potential for advancing the field of neural regeneration, offering innovative solutions to restore neural function and improve the quality of life for patients with neurological injuries or disorders. BCI systems are expected to play a transformative role by bridging damaged neural circuits and facilitating communication between the nervous system and external devices (Fetz 2007). For instance, BCIs could be integrated with neural stimulation techniques to promote the regeneration of damaged neurons and enhance synaptic plasticity (Jackson and Zimmermann 2012). By decoding neural signals and providing real-time feedback, BCIs may enable precise control of limbs, prosthetics, or exoskeletons, aiding in motor recovery for individuals with spinal cord injuries or stroke (Dong et al. 2023). To overcome the fundamental limitations of conventional chronic BCIs, researchers have developed neural dust, an innovative platform enabling long-term, high-density neural recordings (Neely et al. 2018; Patch 2021). This breakthrough technology employs ultrasonic power delivery and backscatter communication to establish a fully wireless, batteryless neural interface system. Thus, neural dust addresses critical challenges of traditional implanted bioelectronics, including device miniaturization, power constraints, and chronic tissue response. Additionally, advanced ML algorithms and AI technologies will further enhance the ability of BCIs to adapt to individual neural patterns, improving their efficacy and usability (Glaser et al. 2020). Beyond motor and sensory restoration, BCIs may also contribute to cognitive rehabilitation by stimulating neural pathways involved in memory and learning (Hampson et al. 2013). Deep brain stimulation (DBS) has emerged as a promising therapeutic intervention for neuropsychiatric conditions, particularly major depressive disorder (MDD) (Alagapan et al. 2023; Scangos et al. 2021). Recent technological frameworks show potential to advance biomarker-driven neural interfaces, potentially improving both mechanistic understanding and treatment outcomes across various neuropsychiatric disorders. However, the substantial interindividual variability in treatment response among MDD patients often leads to inconsistent outcomes in clinical trials. This heterogeneity underscores the need for personalized neuromodulation strategies, particularly closed-loop systems that deliver precisely timed stimuli triggered by real-time detection of pathological neural states. Collectively, BCI technology combined with neural regeneration strategies promises to unlock new frontiers in treating neurological conditions, ultimately paving the way for more effective and personalized therapeutic interventions.

## Conclusions and perspectives

The field of neural regeneration has witnessed remarkable advancements in recent years, driven by the convergence of cutting-edge technologies that offer new possibilities for repairing and restoring damaged neural functions. In addition to the aforementioned technological approaches, rapid progress in genetic engineering, life-omics, viral engineering, electrophysiology, etc., has also enabled researchers to have a new impetus for exploring neural regeneration. The convergence of these technologies has not only deepened our understanding of neural regeneration mechanisms, but also brought hope for treating conditions, such as SCI, optic nerve injury, and neurodegenerative diseases. Looking ahead, the further integration of these multidisciplinary technologies will drive the field toward more precise, efficient, and personalized therapeutic strategies, ultimately transforming the landscape of neural regeneration and improving outcomes for those affected by neurological disorders.

While neural regeneration technologies hold transformative potential, most remain in preclinical development due to several translational challenges. A primary obstacle involves immune responses to viral vector delivery systems, where pre-existing immunity against both viral capsids and cross-reactive serotypes compromises efficacy (Carneiro and Schaffer 2024; Chhabra et al. 2024), necessitating improvements in delivery efficiency and tissue specificity. For CRISPR-Cas9 therapies currently in clinical trials (e.g., for inherited blindness) (Geiger et al. 2025), the permanent nature of genetic modifications demands rigorous safety evaluation, particularly regarding off-target effects. Organoid technology, despite its potential in regenerative medicine and precision therapy (Arjmand et al. 2023; Huang et al. 2025), faces standardization challenges that limit clinical application. Similarly, BCIs encounter biocompatibility issues and mechanical mismatches between electrodes and neural tissue, which provoke immune responses and scarring, ultimately degrading long-term performance (Gao et al. 2025). Addressing these challenges through multidisciplinary innovation will be crucial for developing safe, effective neural regeneration therapies capable of transforming neurological disorder treatment.

## Data Availability

Not applicable.

## Abbreviations

CNS: Central nervous system
3D: Three-dimensional
AI: Artificial intelligence
BCI: Brain-computer interface
SCI: Spinal cord injury
AD: Alzheimer's disease
PD: Parkinson's disease
iPSCs: Induced pluripotent stem cells
CST: Corticospinal tract
GPCRs: G protein-coupled receptors
DREADDs: Designer receptors exclusively activated by designer drugs
RGC: Retinal ganglion cell
DRG: Dorsal root ganglion
2D: Two-dimensional
ES: Electrical stimulation
hNSCs: Human neural stem cells
ALS: Amyotrophic lateral sclerosis
DSBs: Double-stranded breaks
HR: Homologous recombination
NHEJ: Non-homologous end joining
ZFNs: Zinc finger nucleases
TALENs: Transcription activator-like effector nucleases
CRISPR: Clustered regularly interspaced short palindromic repeats
Cas: CRISPR associated protein
OPCs: Oligodendrocyte progenitor cells
MS: Multiple sclerosis
RAGs: Regeneration-associated genes
AAV: Adeno-associated virus
scRNA-seq: Single-cell transcriptome sequencing
ML: Machine learning
GANs: Generative adversarial networks
FNNs: Feedforward neural networks
CNNs: Convolutional neural networks
DBS: Deep brain stimulation
MDD: Major depressive disorder
